# Downregulation of the *Glo1* Gene Is Associated with Reduced Adiposity and Ectopic Fat Accumulation in Spontaneously Hypertensive Rats

**DOI:** 10.3390/antiox9121179

**Published:** 2020-11-26

**Authors:** Jan Šilhavý, Hana Malínská, Martina Hüttl, Irena Marková, Olena Oliyarnyk, Petr Mlejnek, Miroslava Šimáková, František Liška, Ludmila Kazdová, Radka Moravcová, Jiří Novotný, Michal Pravenec

**Affiliations:** 1 Institute of Physiology of the Czech Academy of Sciences, 14220 Prague, Czech Republic; Jan.Silhavy@fgu.cas.cz (J.Š.); Petr.Mlejnek@fgu.cas.cz (P.M.); Miroslava.Simakova@fgu.cas.cz (M.Š.); Frantisek.Liska@lf1.cuni.cz (F.L.); 2 Institute for Clinical and Experimental Medicine, 14021 Prague, Czech Republic; haml@ikem.cz (H.M.); mabw@ikem.cz (M.H.); irma@ikem.cz (I.M.); ooliyarnyk@yahoo.com (O.O.); lukazdova@seznam.cz (L.K.); 3 Institute of Biology and Medical Genetics, First Faculty of Medicine, Charles University and General University Hospital, 12800 Prague, Czech Republic; 4 Department of Physiology, Faculty of Science, Charles University, 12843 Prague, Czech Republic; radka.moravcova@natur.cuni.cz (R.M.); novotnj99@natur.cuni.cz (J.N.)

**Keywords:** *Glo1* gene knockdown, spontaneously hypertensive rat, methylglyoxal, heart, adipose tissue, insulin resistance, AMPK, GLUT4

## Abstract

Methylglyoxal (MG), a potent precursor of advanced glycation end-products (AGE), is increased in metabolic disorders such as diabetes and obesity. MG and other dicarbonyl metabolites are detoxified by the glyoxalase system in which glyoxalase 1, coded by the *Glo1* gene, serves as the rate-limiting enzyme. In this study, we analyzed the effects of *Glo1* downregulation on glucose and lipid metabolism parameters in spontaneously hypertensive rats (SHR) by targeting the *Glo1* gene (SHR-*Glo1*^+/−^ heterozygotes). Compared to SHR wild-type animals, SHR-*Glo1*^+/−^ rats showed significantly reduced *Glo1* expression and lower GLO1 activity in tissues associated with increased MG levels. In contrast to SHR controls, SHR-*Glo1*^+/−^ rats exhibited lower relative weight of epididymal fat, reduced ectopic fat accumulation in the liver and heart, and decreased serum triglycerides. In addition, compared to controls, SHR-*Glo1*^+/−^ rats showed reduced serum insulin and increased basal and insulin stimulated incorporation of glucose into white adipose tissue lipids (lipogenesis). Reduced ectopic fat accumulation in the heart was associated with significantly increased pAMPK/AMPK ratio and GLUT4 activity. These results provide evidence that *Glo1* downregulation in SHR is associated with reduced adiposity and ectopic fat accumulation, most likely mediated by AMPK activation in the heart.

## 1. Introduction

Metabolic disorders such as diabetes and obesity are associated with increased dicarbonyl stress, specifically with increased levels of dicarbonyl toxic metabolites, primarily methylglyoxal (MG), a potent precursor of advanced glycation end-products (AGE). Reactive dicarbonyls are mainly produced during glycolysis, glyceroneogenesis, lipid peroxidation, and glucose auto-oxidation. Dicarbonyl accumulation in tissues can activate inflammatory pathways, increase oxidative stress, impair glucose tolerance, and contribute to the pathogenesis of vascular complications [1,2].

MG and other dicarbonyl metabolites are detoxified by the glyoxalase system, an enzymatic pathway found in the cytosol of cells. This system consists of two enzymes—glyoxalase 1 (GLO1), which is dependent on reduced glutathione (GSH), and glyoxalase 2 (GLO2)—coded by the *Glo1* and *Glo2* genes, respectively. GLO1 is the rate-limiting enzyme of the glyoxalase system. Therefore, reduced GLO1 expression and activity can precipitate the adverse effects of dicarbonyl stress [3]. Impairment to the glyoxalase system is linked to diabetic, cardiovascular, and neurodegenerative disorders. Abnormal activity and expression of GLO1 in various diseases make this enzyme a promising target for drug design [4]. The human GLO1 gene includes the following regulatory elements: E2F4 (early gene 2 factor isoform 4), AP-2α (activating enhancer-binding protein 2α), IRE (insulin response element), MRE (metal response element), and ARE (antioxidant response element) [5]. Therefore, since GLO1 is subject to the regulatory influence of these transcription factors, it potentially not only affects dicarbonyl and oxidative stress, but also lipid and glucose metabolism. To test this theory, we analyzed the metabolic effects of the genetically downregulated *Glo1* gene on dicarbonyl and oxidative stress and on parameters of glucose and lipid metabolism in spontaneously hypertensive rats (SHR). Although the SHR is mainly a model of essential hypertension, under specific environmental conditions, such as when fed a high fructose or folate deficient diets, it also develops disturbances of lipid and glucose metabolism that are typical of metabolic syndrome (e.g., [6,7]). The same is true when the SHR is genetically modified, for instance by transgenic expression of resistin [8] or human gene for C-reactive protein (CRP) [9].

## 2. Materials and Methods

### 2.1. Animals

The SHR/OlaIpcv (referred to as SHR) and SHR-*Glo1^+/−^* knockout heterozygous rats used in the current study were housed in an air-conditioned animal facility and allowed free access to standard laboratory chow and water. Biochemical and metabolic phenotypes in both strains were assessed in 3-month-old non-fasted male rats (*n* = 8 per strain). Rats were killed by decapitation and tissues were collected. All experiments were performed in agreement with the Animal Protection Law of the Czech Republic and approved by the Ethics Committee of the Institute of Physiology, Czech Academy of Sciences, Prague, Czech Republic (71/2015).

### 2.2. Generation of Glo1 Knockout SHR Rats

*Glo1* knockout rats were generated by microinjecting the fertilized ova of SHR rats with a ZFN (Zinc Finger Nuclease) construct from Sigma-Aldrich. The construct was designed to target the first exon using the following sequence of ZFN binding (capital letters) and cutting sites (small letters): TCTGACCCGGATCCAAGCaccaagGGTGGGTGGCAGGGC (NCBI gene accession number 294320). DNA samples isolated from 120 rats born after microinjection with the ZFN construct were amplified using primers flanking the target sequence ZFN F: 5′- TACACTGTGATTGGCTGGGA-3′ and ZFN R: 5′- GATCACTCCACTGCGCC-3′. Altogether, 3 positive animals were detected using Cel-I endonuclease for screening PCR products. SHR-*Glo1*^tm1^/OlaIpcv line 188 (referred to as SHR-*Glo1^+/−^*) with a 15 bp deletion was selected for further analysis. Although SHR-*Glo1^−/−^* homozygotes are fully viable, we chose the SHR-*Glo1^+/−^* heterozygotes for analysis since GLO1 activity is reduced but not totally suppressed in human metabolic disorders. Therefore, SHR-*Glo1^−/−^* homozygotes were excluded given our focus of interest was on the metabolic effects associated with reduced GLO1 activity. 

### 2.3. Glucose Utilization in Epididymal Adipose Tissue

Glucose utilization in adipose tissue was determined ex vivo by measuring the incorporation of radioactive ^14^C-U glucose into adipose tissue lipids. Distal parts of epididymal adipose tissue were rapidly dissected and incubated for 2 h in Krebs–Ringer bicarbonate buffer with 5 mmol/L glucose, 0.1 μCi ^14^C-U glucose/mL (UVVR, Prague, Czech Republic), and 2% bovine serum albumin, with a gaseous phase of 95% O_2_ and 5% CO_2_ in the presence or absence of insulin in incubation media (250 μU/mL). All incubations were performed at 37 °C in sealed vials in a shaking water bath. Estimation of ^14^C-glucose incorporation into neutral lipids was performed as follows: Briefly, adipose tissue was removed from the incubation medium, rinsed in saline, and immediately placed in chloroform. Pieces of tissue were dissolved using a Teflon pestle homogenizer. Methanol was subsequently added (chloroform:methanol 2:1), with lipids extracted at 4 °C overnight. After removing the remaining tissue and adding KH_2_PO_4_, a clear extract was taken for further analysis. An aliquot was evaporated and reconstituted in scintillation liquid, with radioactivity then measured by scintillation counting. 

### 2.4. Tissue Triglyceride and Cholesterol Measurements

To determine triglycerides in the liver and heart, tissues were powdered under liquid N_2_ and extracted for 16 h in chloroform:methanol followed by the addition of 2% KH_2_PO_4_. The solution was then centrifuged. The organic phase was removed and evaporated under liquid N_2_, with the resulting pellet subsequently dissolved in isopropyl alcohol. Triglyceride content was determined by enzymatic assay (Erba-Lachema, Brno, Czech Republic). 

### 2.5. Parameters of Dicarbonyl and Oxidative Stress

Levels of reactive dicarbonyls (MG and GL) in plasma and tissues were determined by high performance liquid chromatography. After derivatization with diamino-dimethoxybenzene, samples were injected in a C18 column (Waters Corporation) with two mobile phases and detected using fluorescence. GLO1 activity was analyzed according to a method described by Arai [10]. Red blood cells were collected by centrifugation of blood (EDTA) samples and washed 3 times with PBS pH 7.4. The washed cells were lysed using cold deionized water. Hemoglobin concentrations were determined using Drabkin’s assay (Sigma-Aldrich, Prague, Czech Republic).

Oxidative stress was measured according to the activities of antioxidant enzymes, concentrations of reduced and oxidized glutathione, and levels of lipoperoxidation products. Activities of superoxide dismutase (SOD), glutathione peroxidase (GSH-Px), and glutathione reductase (GR) were analyzed using Cayman Chemicals assay kits (Ann Arbor, MI, USA). Catalase (CAT) activity measurement was based on the ability of H_2_O_2_ and ammonium molybdate to produce a color complex detected spectrophotometrically. Lipoperoxidation products were assessed based on concentrations of thiobarbituric acid-reactive substances (TBARS) by assaying the reaction with thiobarbituric acid. Concentrations of reduced (GSH) and oxidized (GSSG) forms of glutathione were determined using a HPLC kit with fluorescence detection (ChromSystems, Gräfelfing, Germany).

### 2.6. Biochemical Analysis

Blood glucose levels were measured by glucose oxidase assay (Erba-Lachema, Brno, Czech Republic) from tail vein blood. Serum NEFA (non-esterified fatty acids) levels were determined using an acyl-CoA oxidase-based colorimetric kit (Roche Diagnostics, Mannheim, Germany). Serum triglycerides and total cholesterol concentrations were measured using standard enzymatic methods (Erba-Lachema, Brno, Czech Republic). Serum insulin concentrations were determined using a rat insulin ELISA kit (Mercodia, Uppsala, Sweden). The level of albumin in urine was analyzed by HPLC and UV-VIS detection with adjustment for creatinine concentration. Creatinine concentration was determined using an enzymatic creatinine assay kit (Roche Diagnostics, Mannheim, Germany).

### 2.7. Histological Methods

After sampling, tissue samples were fixed immediately in 4% formaldehyde solution for 48 h and processed in paraffin blocks using standard techniques. Three-to-five-μm-thick slices were cut from each sample using a microtome. The first slices were stained with hematoxylin-eosin (DiaPath, Martinengo, Italy). The prepared slides were then evaluated by a veterinary histopathologist.

### 2.8. Gene Expression Determined by Real-Time PCR

Reverse transcription and quantitative real-time PCR analysis of *Glo1* gene was performed using the TaqMan RNA-to-C_T_ 1-Step Kit, TaqMan Gene Expression Assay (Applied Biosystems, Foster City, CA, USA), and the ViiA^TM^ 7 Real-Time PCR System (Applied Biosystems, Foster City, CA, USA). Relative expression was determined after normalization against β-actin as an internal reference and calculated using the 2^−ΔΔCt^ method.

### 2.9. Determination of GLUT4 and AMPK Activity: Tissue Homogenization and Fractionation

Frozen samples of the heart, liver and white adipose tissue (WAT) were obtained from 6 animals in each group. The samples were placed in 4 volumes of ice-cold TMES buffer (20 mM Tris, 3 mM MgCl_2_, 1 mM EDTA and 0.25 M sucrose; pH 7.4) containing protease and phosphatase inhibitors (cOmplete Protease Inhibitor Cocktail and PhosSTOP; Roche Diagnostics, Basel, Switzerland), cut into small pieces, and homogenized in an ice-bath with a Potter-Elvehjem homogenizer (20 strokes at 1200 rpm) on ice. The resulting suspension was centrifuged at 2000× *g* for 10 min (4 °C) to remove large tissue debris and nuclei. The resulting post-nuclear supernatant (PNS) was centrifuged at 33,000× *g* for 20 min (4 °C) to isolate crude plasma membranes (CPM). The pelleted membranes were re-suspended in half of the initial volume of TMES buffer containing 1% Nonidet P-40. The supernatant was centrifuged at 200,000× *g* for 60 min to isolate the light microsomal fraction (LM). The pellet was then re-suspended in half of the initial volume of TMES buffer containing 1% Nonidet P-40. Aliquots of CPM and LM were snap-frozen and stored at −80 °C.

### 2.10. Determination of GLUT4 and AMPK Activity: Electrophoresis and Western Blotting

Samples of CPM and LM were solubilized in Laemmli buffer. For SDS-PAGE, equal volumes of samples (10 μL per lane) were loaded on standard 10% polyacrylamide gels. After electrophoresis, the resolved proteins were transferred to a nitrocellulose membrane (Protran BA85, GE Healthcare, Little Chalfont, Buckinghamshire, UK), blocked with 5% non-fat dry milk in TBS buffer (10 mM Tris and 150 mM NaCl; pH 8.0) for 30 min, and then incubated with specific primary antibodies overnight at 4 °C. The membrane was subsequently washed three times for 10 min with TBS buffer containing 0.3% Tween 20 (TBS-T) before being reacted with an appropriate secondary antibody conjugated to horseradish peroxidase. After a 1-h incubation, the blots were washed with TBS-T and visualized using enhanced chemiluminescence (ECL) with SuperSignal West Dura substrate (Pierce Biotechnology, Rockford, IL, USA). ECL signals were detected by exposure to standard X-ray film (Agfa). Film was digitized using a high resolution CCD scanner (EPSON Perfection V600 Photo). Images were quantitatively analyzed using ImageJ software (University of Wisconsin, Madison, WI, USA).

### 2.11. Statistical Analysis

All data are expressed as means ± S.E.M. We used the *t*-test or rank-sum test for two-group comparisons. Statistical significance was defined as *p* < 0.05.

## 3. Results

### 3.1. Glyoxalase 1 Gene Expression, Enzyme Activity, and Dicarbonyl and Oxidative Stress

**Figure 1:**
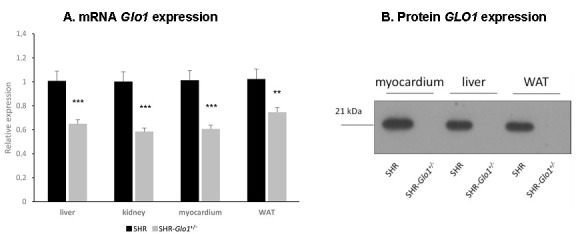
Relative mRNA expression of the *Glo1* gene and protein levels of GLO1 in respective tissues of SHR-*Glo1^+/−^* heterozygotes and SHR wild-type rats. Quantitative real-time PCR analysis (**A**) and western blot detection (**B**) of *Glo1* were performed in tissue homogenates as described in Materials and Methods. Results expressed as the mean ± S.E.M. were compared by one-way ANOVA (**, *p* < 0.01; ***, *p* < 0.001). WAT = white adipose tissue.

In the current study, we chose the SHR-*Glo1^+/−^* heterozygotes for analysis since GLO1 activity in human metabolic disorders is reduced but not totally suppressed. Therefore, SHR-*Glo1^−/−^* homozygotes (though fully viable) were not used given our focus of interest was on the metabolic effects associated with reduced GLO1 activity. Expression of the *Glo1* gene in the liver, heart, renal cortex and white adipose tissue (WAT) of SHR-*Glo1^+/−^*rats was approximately 30–40% lower compared to SHR wild-type controls (Figure 1A). Figure 1B shows strong reduction in GLO1 protein in SHR-*Glo1^+/−^* rats. As shown in Table 1, reduced expression of *Glo1* in SHR-*Glo1^+/−^* heterozygotes was associated with significantly reduced activity of GLO1 in the liver, heart and erythrocytes. We observed reduced GLO1 activity in epididymal adipose tissue but the difference was not statistically significant. Reduced activity of GLO1 in SHR-*Glo1^+/−^* heterozygotes was associated with significantly increased dicarbonyl stress. MG and GL concentrations significantly increased in the blood and heart, but no significant differences were observed in the kidney, liver or epididymal adipose tissue (Table 1). 

Downregulation of *Glo1* was associated with significantly increased oxidative stress in the liver and kidney cortex when lipoperoxidation products measured as TBARS and SOD activity were increased in SHR-*Glo1^+/−^* rats compared to wild-type SHR (Table 2). Reduced levels of glutathione, which indicate oxidative as well as dicarbonyl stress, were observed in all tissues except for the heart (Table 1). However, we observed no significant changes in the activity of glutathione-dependent antioxidant enzymes (Table 2).

### 3.2. Parameters of Glucose and Lipid Metabolism

Table 3 shows that SHR-*Glo1^+/−^* heterozygotes in comparison to wild-type SHR exhibited lower body weight, decreased relative weight of epididymal fat, reduced ectopic lipid accumulation in the liver and heart, and lower concentrations of serum triglycerides. In addition, SHR-*Glo1^+/−^* heterozygotes compared to SHR exhibited ameliorated insulin resistance when they had lower serum insulin levels and their adipose tissue was more sensitive to insulin action, as indicated by increased insulin-stimulated lipogenesis (Table 3).

### 3.3. Effect of Glo1 Downregulation on GLUT4 and AMPK Activation

**Figure 2:**
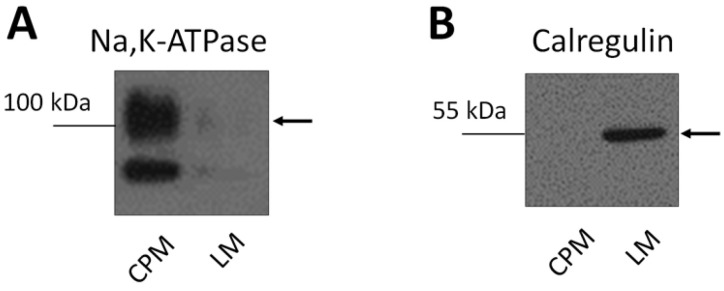
Distribution of Na,K-ATPase and calregulin (markers of the plasma membrane and endoplasmic reticulum, respectively) in myocardial crude plasma membranes (CPM) and light microsomal membranes (LM). CPM and LM were prepared by fractionation of the left ventricles from SHR and SHR-*Glo1^+/−^* rats and equal sample volumes (10 μL of each fraction) were subjected to electrophoresis and immunoblotted with antibodies against Na,K-ATPase (**A**) and calregulin (**B**) as described in Materials and Methods. Typical immunoblots are shown.

**Figure 3:**
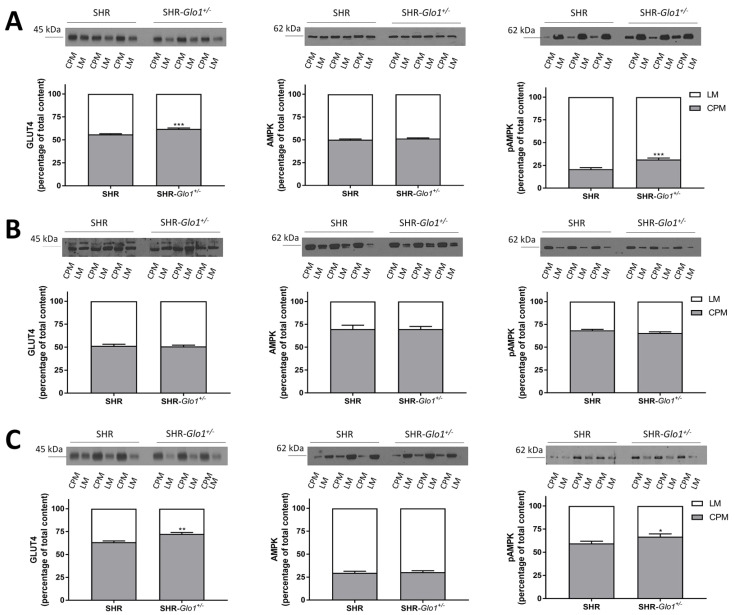
Distribution of GLUT4, AMPK, and pAMPK in crude plasma membranes (CPM) and light microsomal membranes (LM). CPM and LM were prepared by fractionation of the left ventricles (**A**), liver (**B**) and white adipose tissue (**C**) from SHR and SHR-*Glo1^+/−^* rats. Equal sample volumes were subjected to electrophoresis and immunoblotted with antibodies against GLUT4, AMPK, and pAMPK as described in Materials and Methods. The most typical immunoblots are shown on top. Protein expression levels were determined based on 3 separate experiments using individual samples from 6 animals in each group. The proportions of proteins distributed in CPM and LM are expressed as the mean ± S.E.M. Results were compared by one-way ANOVA followed by Bonferroni’s post-hoc test (*, *p* < 0.05; **, *p* < 0.001; *** *p* <0.001).

**Figure 4:**
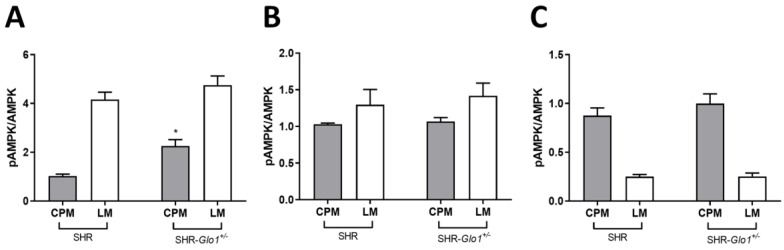
The pAMPK/AMPK ratio in crude plasma membranes (CPM) and light microsomal membranes (LM) prepared by fractionation of the left ventricles (**A**), liver (**B**) and white adipose tissue (**C**) from SHR and SHR-*Glo1^+/^*rats. Values are the mean ± S.E.M. of three separate experiments (*, *p* < 0.05 vs. corresponding membrane fraction).

Expression levels of GLUT4 and AMPK were assessed by western blotting in the postnuclear supernatant (PNS), crude plasma membranes (CPM) and light microsomal membranes (LM) prepared from heart, liver and white adipose tissue of SHR and SHR-*Glo1^+/−^* rats. We analyzed CMP and LM fractions because insulin-stimulated transport of glucose into tissues is mediated by the redistribution of the insulin-responsive glucose transporter GLUT4 from intracellular GLUT4 storage vesicles to the plasma membrane (CPM) [11]. AMPK phosphorylation may also be involved in GLUT4 translocation and glucose uptake in muscle, in 3T3L1 adipocytes and in adipose tissue [12,13,14]. The distribution of Na,K-ATPase (plasma membrane marker) and calregulin (endoplasmic reticulum marker) in membrane fractions enriched in CPM and LM is shown in Figure 2. A distinct pattern in the distribution of these markers between CPM and LM indicated that the two fractions had been successfully separated. No significant differences were observed in the total amount of GLUT4 and AMPK in PNS from SHR and SHR-*Glo1^+/−^* rats (Supplementary Figure S1). However, the proportion of GLUT4 was significantly increased in CPM from heart and white fat of SHR-*Glo1^+/−^* when compared to SHR (Figure 3A,C, Supplementary Figure S2). Interestingly, downregulation of *Glo1* did not affect the distribution of GLUT4 in CPM and LM from the liver (Figure 3A, Supplementary Figure S2). Equal loading of respective samples was checked using Ponceau S staining (Supplementary Figure S3). The distribution of AMPK between CPM and LM was not affected by *Glo1* downregulation in either tissue. However, the level of phosphorylation of AMPK was significantly increased in CPM from heart and white fat of SHR-*Glo1^+/−^* when compared to SHR (Figure 3A,C). The distribution of phosphorylated AMPK (pAMPK) remained unchanged in CPM and LM from the liver after *Glo1* downregulation (Figure 3B). The ratio of pAMPK to total AMPK in cardiac CPM of SHR-*Glo1^+/−^* rats was twofold higher than that of SHR rats (Figure 4A). Downregulation of *Glo1* apparently did not induce any significant change in the pAMPK/AMPK ratio in CPM from the liver or white fat and in LM prepared from either tissue (Figure 4). Nevertheless, there was an upward trend (a non-significant increase by 14%) in the pAMPK/AMPK ratio in white fat CPM of SHR-*Glo1^+/−^* rats, compared to SHR rats. Interestingly, while the phosphorylation level of AMPK in white fat CPM was markedly higher than in LM, the opposite was true for the distribution of pAMPK in cardiac membrane fractions. This may partially explain why the small but significant increase observed in the proportion of pAMPK in white fat CPM after *Glo1* downregulation was reflected only by a tendency towards increased pAMPK/AMPK ratio in this membrane fraction.

### 3.4. Microalbuminuria and Histological Analysis

Compared to SHR, SHR-*Glo1^+/−^* heterozygotes exhibited increased albuminuria (33.62 ± 0.21 vs. 20.09 ± 4.29 mg albumin/g creatinine, *p* = 0.03), despite comparable histological findings in both strains (data not shown) indicating incipient nephropathy.

## 4. Discussion

In this study, we analyzed the metabolic effects of *Glo1* gene downregulation in SHR-*Glo1^+/−^* heterozygotes compared to wild-type SHR controls. The SHR-*Glo1^+/−^* heterozygotes showed reduced *Glo1* mRNA levels in organs by approximately 30–40%, however, practically zero GLO1 protein concentrations. However, it is possible that western blot analysis was not sensitive enough to detect smaller concentrations of GLO1 protein that would be still sufficient for normal GLO1 activity. While we observed an association of reduced *Glo1* expression with increased dicarbonyl stress in the blood and heart, no significant differences in concentrations of dicarbonyl compounds were observed in the kidney, liver or epididymal adipose tissue. It should be noted that in addition to GLO1, MG is detoxified by other enzymes such as aldo-keto reductases (AKRs) and it has been reported that AKRs can effectively compensate for reduced GLO1 activity in C57BL/6-*Glo1^−/−^* mice to prevent the accumulation of MG [15]. Accordingly, it is possible that the lack of correlation between changes in GLO1 activity and MG levels in tissues is at least partially due to compensatory effects of AKRs on MG detoxification. The most pronounced metabolic effects of the *Glo1* targeted gene were observed in visceral adipose tissue and in ectopic fat accumulation in heart and liver. Endogenous MG in adipose tissue is produced mainly by glyceroneogenesis during triglyceride synthesis [1]. Targeted *Glo1* gene was associated with reduced fat mass, suggesting less of a demand for glycerol-3-phosphate in triglyceride synthesis. It is possible, therefore, that a reduction in the glyceroneogenesis rate leads to decreased MG production. This may also explain why we found only a non-significantly reduced GLO1 activity and increased MG levels in adipose tissue in SHR-*Glo1^+/−^* compared to SHR rats. In addition, SHR-*Glo1^+/−^* rats exhibited amelioration of insulin resistance in adipose tissue when insulin-stimulated glucose incorporation into lipids (lipogenesis) was significantly higher. It has been recently demonstrated in WAT that lipogenesis, when combined with triglyceride/fatty acid cycling activity, contributes to a lean phenotype in mice [16]. This increased glucose uptake was caused by a significantly higher proportion of the GLUT4 glucose transporter on the adipocyte surface (CPM), however, the pAMPK/AMPK ratio in SHR-*Glo1^+/−^* compared to SHR wild-type rats remained similar. On the other hand, *Glo1* dowregulation in the heart was associated with higher MG levels and increased localization of GLUT4 to the CPM fraction; the concomitant increase in the pAMPK/AMPK ratio suggests that AMPK activation may be involved in the observed partial GLUT4 translocation. Discrepancy between changes in pAMPK/AMPK ratio in adipose tissue versus heart might be due to tissue-specific differences in AMPK function. For instance, there was reported a significant organ specificity in gene expression changes induced in mice by either exercise or direct pharmacological activation of AMPK [17].

The mechanisms connecting *Glo1* downregulation with AMPK activation are not known and go beyond the scope of this study. Hypothetically, MG can stimulate AMPK activation via direct or indirect pathways. MG can increase the AMP-to-ATP ratio, a stimulus for AMPK, by decreasing the mitochondrial membrane potential and intracellular ATP levels [18,19,20]. AMPK can also be activated by oxidative stress via the classical AMP-mediated pathway [21]. Finally, it is also possible that MG activates AMPK directly [22].

Only a few studies in animal models reported analyses of *Glo1* manipulation or MG administration on the outcome of experimental heart pathologies. For instance, overexpression of *Glo1* in C57BL/6 transgenic mice was associated with reduced MG AGE levels and the mice exhibited superior cardiac function at 4 weeks post-MI (myocardial infarction) compared to wild-type mice [23]. The same *Glo1* transgenic mice were treated with streptozotocin (STZ) to induce type 1 diabetes. When compared to wild-type controls also treated with STZ, *Glo1* overexpression delayed and limited the loss of cardiac function [24]. Wistar rats were submitted to MG chronic administration for 3 months with gradually enhanced concentration, up to 75 mg/kg. It was reported that such treatment mimicked most diabetic alterations, similar to those observed in Goto–Kakizaki (GK) rats, a model of non-obese type 2 diabetes [25]. These studies provided evidence that dicarbonyl stress has adverse effects on cardiac function. 

The effects of *Glo1* downregulation on metabolic parameters were studied in C57BL/6 mice that were derived by transgenesis with a hairpin RNA sequence targeting the *Glo1* gene. Compared to wild-type controls, these knockdown mice exhibited significantly reduced GLO1 activity in all of the organs analyzed including heart. However, no elevation in levels of methylglyoxal-derived AGEs was reported, nor were there any significant differences in body weight irrespective of standard or high-fat diets; energy expenditure was found to be similar to controls [26]. In another study, transgenic mice overexpressing the human GLO1 gene showed no differences in body weight, fasting plasma glucose, total cholesterol, triglycerides, or insulin and leptin concentrations [27]. In addition, C57BL/6-*Glo1^−/^^−^* mice exhibited similar levels of MG to wild-type controls and showed no sign of nephropathy after treatment with STZ [15]. On the other hand, in vitro experiments demonstrated that *Glo1* knockdown by siRNA in L6 myoblasts induced intracellular accumulation of MG. This effect was associated with augmented GLUT4 levels on cell surfaces, resulting in increased glucose uptake independent of insulin signaling [28].

Treatment of cultured cells and rodent models with exogenous MG have yielded discrepant results compared to the metabolic effects associated with increased levels of endogenous MG in models with the downregulated *Glo1* gene. Riboulet-Chavey et al. [29] found that insulin signaling was inhibited in short-term MG-exposed L6 myoblasts, resulting in impaired glucose uptake. It has been reported that MG treatment inhibited the insulin receptor pathway in 3T3L1 adipocytes [30,31] and pancreatic cell line INS-1E [32]. One study to treat hereditary hypertriglyceridemic rats (a model of dyslipidemia and insulin resistance) with intragastric administration of MG (0.5 mg/kg body weight for 4 weeks) reported a development in dicarbonyl and oxidative stress associated with reduced glucose tolerance and renal microvascular complications [33]. Another study showed that Sprague–Dawley rats treated with 1% MG in drinking water for 4 weeks were predisposed to diabetes and salt-sensitive hypertension [34]. Similar results were observed in Sprague–Dawley rats, where MG infusion (60 mg/kg/day) for 4 weeks was associated with impaired glucose tolerance, reduced levels of the GLUT-4 glucose transporter, and decreased insulin-stimulated glucose uptake in adipose tissue [30]. Similarly, mice treated with MG (50–75 mg/kg, daily, i.p.) for 7 weeks developed insulin resistance [35]. On the other hand, it has been found that long-term MG intake (200–300 mg/kg BW/day) in C57BL/6 mice from 6 months till death had no toxic effects. At 24 months of age, these mice showed no changes in GLO1 activity in blood or tissues and no signs of renal insufficiency or diabetes; AGE modifications of plasma and vessel proteins remaining unchanged. It has been suggested that dietary MG is mainly detoxified through renal excretion [36]. In summary, increased MG concentrations observed in rodent models with the downregulated *Glo1* gene are associated with no or favorable metabolic effects, whereas administration of exogenous MG is associated with adverse metabolic effects. The discrepancies in these results may be attributed to differences between moderately increased concentrations of endogenous MG in models with genetic downregulation of the *Glo1* gene and the much higher MG levels in most rodent models treated with exogenous MG given in drinking water, by gavage, intraperitoneal injections or micropump infusion. The physiological relevance of the latter models is questionable, since they involve the extracellular addition of high MG levels. Not only does exogenous MG have to first pass the cell membrane in order to be detoxified by intracellular enzymes, the extracellular enzymes that metabolize MG have yet to be identified.

The extent of ectopic fat accumulation appears to be significantly linked to visceral adipose tissue [37], which is considered as a useful marker of cardiovascular and metabolic risk [38]. Interestingly, fatty acid incorporation, lipolysis, and release are approximately twofold higher in epicardial adipose tissue (EAT) than in other adipose depots [39]. It is noteworthy that obesity, diabetes as well as heart failure are associated with impaired glucose and lipid uptake in dysfunctional EAT [40,41,42]. EAT is characterized by elevated lipogenesis under these pathological conditions [39]. Here, we observed a decrease in epididymal fat and EAT after *Glo1* downregulation. It is hypothetically possible that increased MG levels in hearts from SHR-*Glo1^+/−^* rats activate AMPK, which reduces ectopic fat accumulation by promoting catabolic processes instead of lipid storage in the heart. 

The results of our study, specifically effects of MG on AMPK and GLUT4 activation in the heart, point to the possible physiological role of endogenous intracellular MG in the regulation of energy metabolism in cardiac tissue. We contend that a physiological increase in endogenous MG during glycolysis activates AMPK and reduces ectopic fat accumulation as a consequence. Similarly, it could be speculated that endogenous MG produced during glyceroneogenesis in WAT may act as a feedback loop in adiposity regulation; however, the increases in MG levels and AMPK activation were not statistically significant in WAT.

Moranu et al. [43] reported the physiological role of endogenous MG in the regulation of energy metabolism in *Drosophila Glo1^KO^* knockouts exhibiting insulin resistance, obesity, and hyperglycemia. It has also been suggested that MG might serve as a cellular proxy for unbalanced DHAP/G3P (dihydroxyacetone phosphate/glyceraldehyde 3-phosphate) flux while normalizing DHAP/G3P concentrations by reducing insulin signaling and increasing biosynthesis of triglycerides. Lending further support for the physiological role of endogenous MG, *Glo1^KO^* flies have been shown to have significantly extended lifespans, as have *C. elegans* worms exhibiting mildly elevated MG levels [44]. Taken together, these studies provide compelling evidence that MG is not just a toxic metabolite but that physiological concentrations of endogenous MG play a role in regulating glucose and lipid metabolism.

## 5. Conclusions

*Glo1* gene targeting in the SHR was associated with dicarbonyl and oxidative stress in some tissues and significant metabolic effects, reduced adiposity and ectopic fat accumulation in the heart, which was associated with AMPK activation. In summary, the *Glo1* gene plays an important role in the detoxification of dicarbonyl compounds and in the regulation of lipid and glucose metabolism by modulating levels of endogenous MG, a potential direct or indirect AMPK activator.

## Figures and Tables

**Table 1:** Parameters of dicarbonyl stress in plasma and tissues of wild-type SHR versus SHR-*Glo1^+/−^* rats with targeted *Glo1* gene.

Trait	SHR	SHR-*Glo1^+/−^*
**Plasma or Blood**		
Glyoxal (nmol/mL) (plasma)	0.097 ± 0.009	0.157 ± 0.012 *
Methylglyoxal (nmol/mL) (plasma)	0.291 ± 0.011	0.726 ± 0.048 **
GSH (μmol/L) (blood)	778.8 ± 5.8	718.1 ± 16.5 *
GSSG (μmol/L) (blood)	53.8 ± 4.9	53.9 ± 8.5
GSH/GSSG ratio	14.9 ± 1.2	13.6 ± 1.6
*Glo1* activity (μmol/min/μg Hb) (erytrocytes)	0.406 ± 0.019	0.192 ± 0.007 **
**White Adipose Tissue**		
Glyoxal (nmol/mg)	0.165 ± 0.003	0.199 ± 0.019
Methylglyoxal (nmol/mg)	2.159 ± 0.301	2.567 ± 0.186
*Glo1* activity (μmol/min/μg protein)	0.831 ± 0.070	0.707 ± 0.069
**Myocardium**		
Glyoxal (nmol/mg)	0.380 ± 0.019	0.960 ± 0.074 **
Methylglyoxal (nmol/mg)	1.054 ± 0.170	2.159 ± 0.102 **
GSH (μmol/mg protein)	12.2 ± 0.8	10.1 ± 0.7
GSSG (μmol/mg protein)	2.8 ± 0.3	2.3 ± 0.2
GSH/GSSG ratio	4.5 ± 0.7	4.4 ± 0.2
*Glo1* activity (μmol/min/μg protein)	0.685 ± 0.055	0.453 ± 0.061 *
**Kidney**		
Glyoxal (nmol/mg)	0.626 ± 0.017	0.717 ± 0.009
Methylglyoxal (nmol/mg)	2.206 ± 0.179	2.114 ± 0.105
GSH (μmol/mg protein)	42.1 ± 2.2	27.0 ± 1.5 **
GSSG (μmol/mg protein)	4.3 ± 0.3	3.2 ± 0.1 **
GSH/GSSG ratio	9.9 ± 1.5	8.6 ± 0.6
**Liver**		
Glyoxal (nmol/mg)	0.728 ± 0.095	0.772 ± 0.095
Methylglyoxal (nmol/mg)	3.093 ± 0.179	3.314 ± 0.371
GSH (μmol/mg protein)	93.5 ± 6.1	68.6 ± 1.1 **
GSSG (μmol/mg protein)	4.7 ± 0.1	4.7 ± 0.2
GSH/GSSG ratio	19.8 ± 0.9	15.0 ± 0.6 **
*Glo1* activity (μmol/min/μg protein)	0.562 ± 0.021	0.285 ± 0.011 **

* and ** denote *p* < 0.05 and *p* < 0.005, respectively; GSH = reduced glutathione; GSSG = oxidized glutathione.

**Table 2:** Parameters of oxidative stress in tissues of wild-type SHR versus SHR-*Glo1^+/−^* rats with targeted *Glo1* gene.

Trait	SHR	SHR-*Glo1*^+/−^
**Myocardium**		
SOD (U/mg protein)	0.075 ± 0.006	0.054 ± 0.004 **
GSH-Px (μM NADPH/min/mg protein)	131 ± 12	138 ± 11
GR (μM NADPH/min/mg protein)	125 ± 8	114 ± 8
CAT (mM H_2_O_2_/min/mg protein)	594 ± 26	530 ± 31
TBARS (nM/mg protein)	0.561 ± 0.026	0.651 ± 0.021 *
**Kidney cortex**		
SOD (U/mg protein)	0.024 ± 0.002	0.026 ± 0.002
GSH-Px (μM NADPH/min/mg protein)	73 ± 6	68 ± 7
GR (μM NADPH/min/mg protein)	28 ± 3	39 ± 5
CAT (mM H_2_O_2_/min/mg protein)	451 ± 26	430 ± 27
TBARS (nM/mg protein)	0.338 ± 0.021	0.391 ± 0.008 *
**Liver**		
SOD (U/mg protein)	0.142 ± 0.009	0.115 ± 0.008 *
GSH-Px (μM NADPH/min/mg protein)	292 ± 17	245 ± 20
GR (μM NADPH/min/mg protein)	139 ± 13	154 ± 23
CAT (mM H_2_O_2_/min/mg protein)	1515 ± 126	1707 ± 154
TBARS (nM/mg protein)	1.759 ± 0.173	1.867 ± 0.128

* and ** denote *p* < 0.05 and *p* < 0.005, respectively; SOD = superoxide dismutase; GSH-Px = glutathione peroxidase; GR = glutathione reductase; CAT = catalase; TBARS = thiobarbituric acid-reactive substances.

**Table 3:** Body weight, relative organ weight, and parameters of glucose and lipid metabolism in wild-type SHR versus SHR-*Glo1^+/−^* rats with reduced expression of the *Glo1* gene.

Trait	SHR	SHR-*Glo1^+/−^*
Body weight (g)	362 ± 4	344 ± 6 *
Relative weight of epididymal fat (g/100 g BW)	0.98 ± 0.02	0.88 ± 0.03 *
Relative liver weight (g/100 g BW)	3.24 ± 0.04	3.11 ± 0.03 *
Non-fasting glucose (mmol/L)	6.3 ± 0.2	6.2 ± 0.1
Insulin (μmol/L)	0.275 ± 0.023	0.188 ± 0.017 *
NEFA (mmol/L)	0.38 ± 0.02	0.42 ± 0.02
Serum triglycerides (mmol/L)	0.48 ± 0.03	0.34 ± 0.01 *
Serum cholesterol (mmol/L)	1.17 ± 0.04	1.10 ± 0.03
Triglycerides in the liver (μmol/g)	6.1 ± 0.5	4.1 ± 0.4 **
Cholesterol in the liver (μmol/g)	10.2 ± 0.5	7.6 ± 0.3 **
Triglycerides in the myocardium (μmol/g)	0.56 ± 0.08	0.32 ± 0.06 *
Basal lipogenesis (nmol glucose/g/2 h)	354 ± 12	505 ± 34 **
Insulin-stimulated lipogenesis (nmol glucose/g/2 h)	734 ± 37	1123 ± 73 **

* and ** denote *p* < 0.05 and *p* < 0.005, respectively; BW = body weight; NEFA = non-esterified fatty acids.

## Supplementary Materials

The following are available online at https://www.mdpi.com/2076-3921/9/12/1179/s1, Figure S1. Distribution of Na/K-ATPase and calregulin (markers of the plasma membrane and endoplasmic reticulum, respectively) in myocardial crude plasma membranes (CPM) and light microsomes (LM). Supplementary Figure S2. Effect of *Glo1* downregulation on GLUT4 expression level in crude plasma membranes (CPM) and light microsomal membranes (LM) prepared by fractionation of the left ventricles (A), liver (B) and white fat tissue (C) from SHR and SHR-*Glo1^+/−^* rats. Supplementary Figure S3. Monitoring of equal loading and transfer efficiency by Ponceau S total protein staining. Samples of CPM and LM (10 μL per lane) prepared by fractionation of the left ventricles (A), liver (B) and white adipose tissue (C) from SHR and SHR-*Glo1^+/−^* rats were subjected to SDS-PAGE and electrotransferred onto nitrocellulose membranes.

## Abbreviations

AGE	Advanced glycation end-products
AMPK	AMP-activated protein kinase
CAT	Catalase
CPM	Crude plasma membranes
GL	Glyoxal
GLO1	Glyoxalase 1
GLUT4	Glucose transporter 4
GSH	Reduced glutathione
GSH-Px	Glutathione peroxidase
GSSG	Oxidized glutathione
LV	Light vesicles
MG	Methylglyoxal
PNS	Postnuclear supernatant
RAGE	Receptor for advanced glycation end-products
SOD	Superoxide dismutase
TBARS	Thiobarbituric acid reactive substances
WAT	White adipose tissue
