# Supplementary material for: Downregulation of the Glo1 Gene Is Associated with Reduced Adiposity and Ectopic Fat Accumulation in Spontaneously Hypertensive Rats
Source: Antioxidants (Basel). 2020 Nov 26;9(12):1179. doi: 10.3390/antiox9121179 (PMC7759780; doi:10.3390/antiox9121179)

Supplementary Figure S1. Western blot analysis of PNS from the left ventricles (A), liver (B) and white adipose tissue (C) obtained from SHR and SHR-Glo1$^{+/-}$ rats. After SDS-PAGE (10 mg of total protein per lane), the proteins were visualized with GLUT4, AMPK and pAMPK antibodies as described in Materials and Methods. Typical immunoblots are shown (at the top). The relative proteins levels (mean ± S.E.M.) determined in 3 separate experiments using individual samples from 6 animals in each group are plotted in arbitrary units (a.u.). Results were compared by one-way ANOVA followed by Bonferonni´s post hoc test.

A – Heart tissue samples

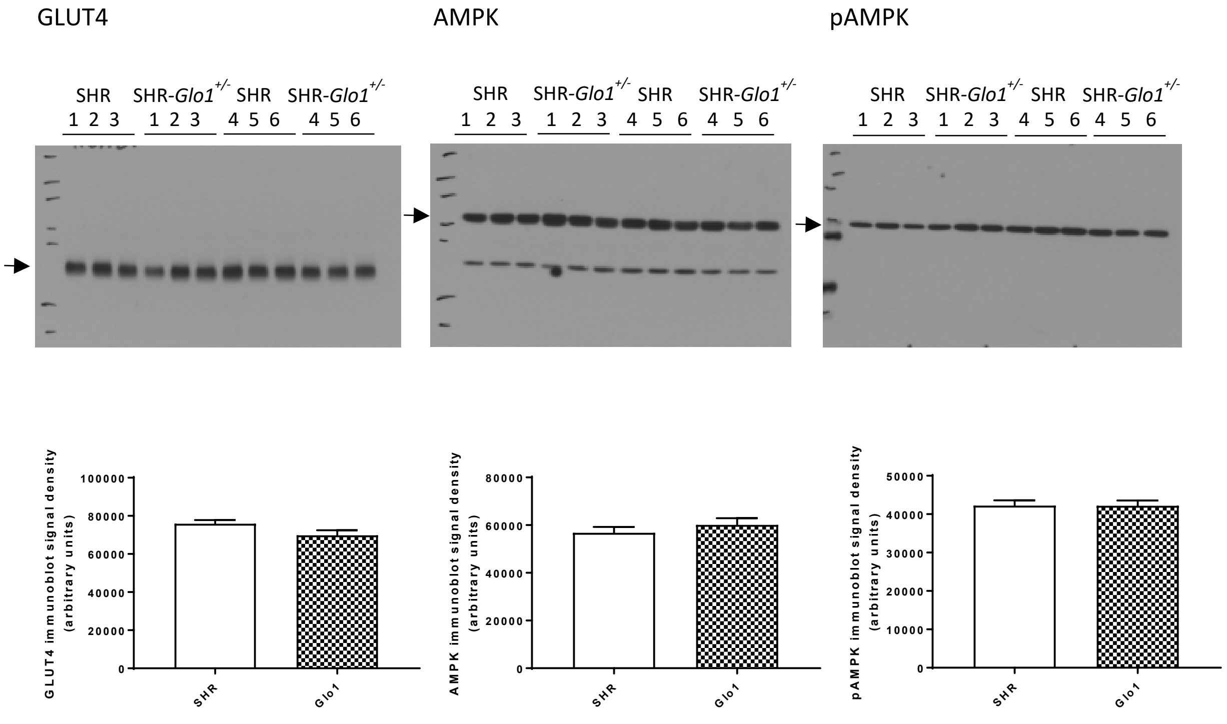

B – Liver tissue samples

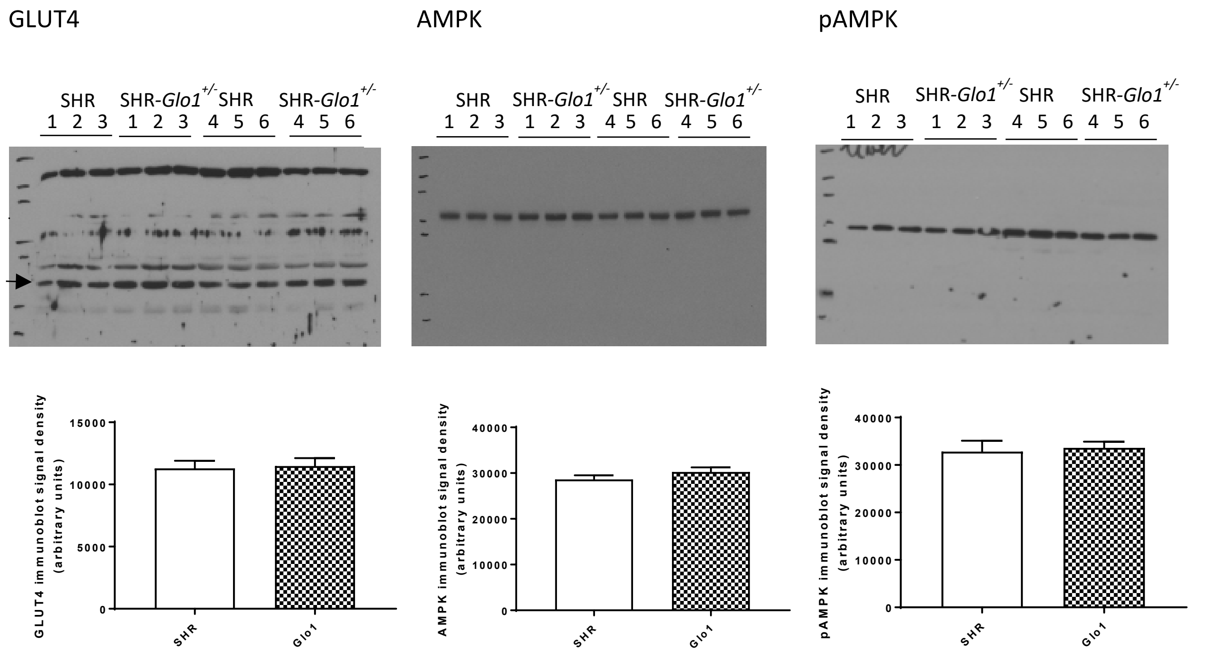

C – Fat tissue samples

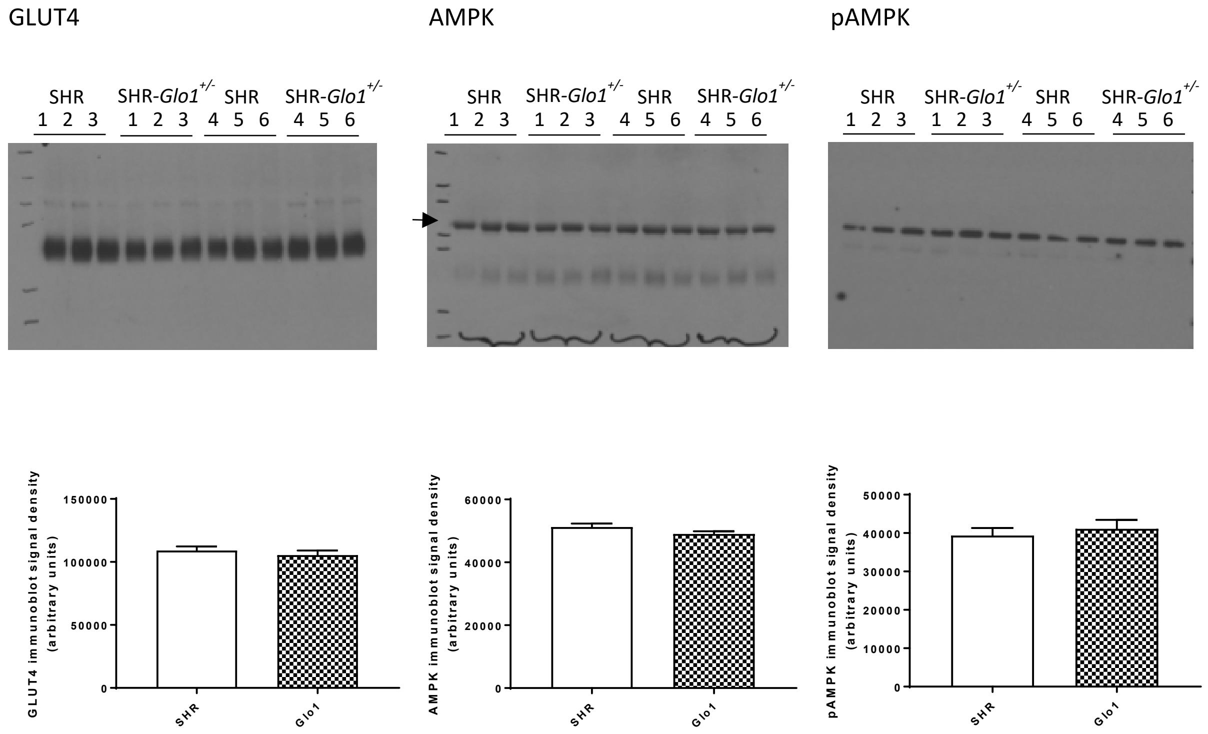

Supplementary Figure S2. Effect of *Glo1* downregulation on GLUT4 expression level in crude plasma membranes (CPM) and light microsomal membranes (LM) prepared by fractionation of the left ventricles (A), liver (B) and white fat tissue (C) from SHR and SHR-Glo$^{1+/-}$ rats. Equal sample volumes were subjected to electrophoresis and immunoblotted with antibodies against GLUT4 as described in Materials and Methods. The relative GLUT4 levels (mean ± S.E.M.) determined in 3 separate experiments using individual samples from 6 animals in each group are plotted in arbitrary units (a.u.). Protein expression levels in CPM and LM in samples from SHR and SHR-Glo$^{1+/-}$ rats were compared by one-way ANOVA followed by Bonferroni's post-hoc test (***, p<0.001 vs CPM; $^{\#}$, p<0.05; $^{\#\#\#}$p<0.001 vs SHR).

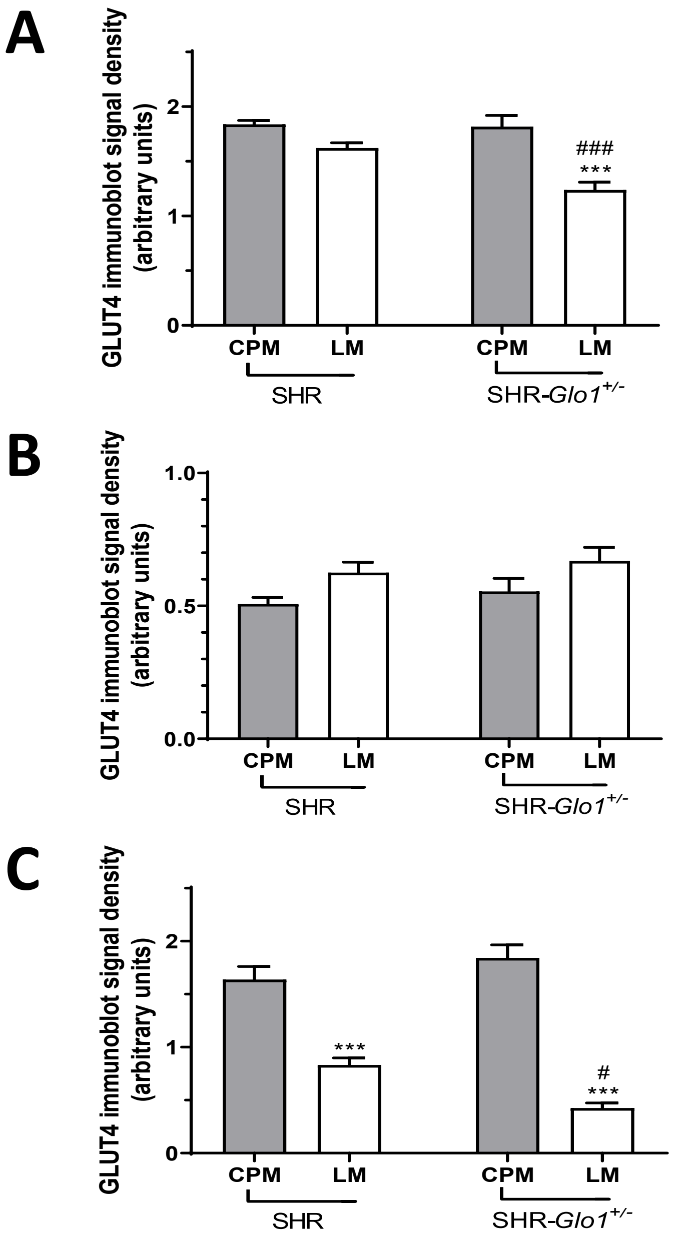

Supplementary Figure S3. Monitoring of equal loading and transfer efficiency by Ponceau S total protein staining. Samples of CPM and LM (10 μl per lane) prepared by fractionation of the left ventricles (A), liver (B) and white adipose tissue (C) from SHR and SHR-Glo1$^{+/-}$ rats were subjected to SDS-PAGE and electrotransferred onto nitrocellulose membranes. After washing in water (three times for 5 min), the membranes were stained for 1 min with Ponceau S stain solution and then briefly destained with several changes of water (about 30 sec each). Subsequently, a permanent record of the staining pattern was made by photocopying the blots. Optical density of protein bands in each lane was determined by using densitometric software (ImageJ). The total amount of protein in each lane (corresponding to individual samples) was used to normalize the abundance of the protein of interest. Nevertheless, there were no significant differences between the results obtained before and after normalization. Densitometric analysis of Ponceau S-stained blots indicated that there were no significant differences between loading of respective samples. Optical densities of the total protein load in the individual lanes as well as average optical densities (± S.E.M.) of the total protein load in the corresponding samples are shown in tables next to the respective images.

A – Heart tissue samples

GLUT4

Average optical density

SHR　　SHR-*Glo1*$^{+/-}$

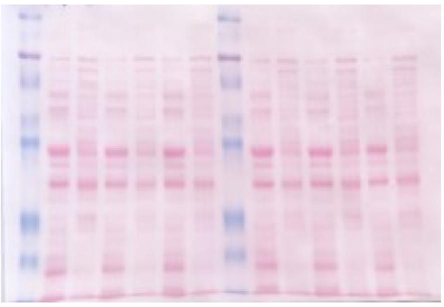

1 2 3 4 5 6 7 8 9 10 11 12

Sample number	Optical density
1	39832.58
2	36556.57
3	40024.73
4	38664.54
5	43495.2
6	36460.08
7	43957.37
8	35228.25
9	46315.56
10	37269.81
11	43932.2
12	38761.13

	SHR	SHR-Glo1$^{+/-}$
CPM	41117.50 ± 1683.11	44735.04 ± 1117.64
LM	37200.40 ± 1017.21	37086.40 ± 1448.11

AMPK

SHR　　SHR-*Glo1*$^{+/-}$

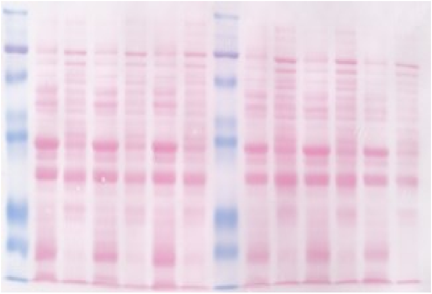

1 2 3 4 5 6 7 8 9 10 11 12

Sample number	Optical density
1	59436.62
2	47612.01
3	58626.86
4	46547.47
5	59136.17
6	47311.15
7	55820.84
8	60995.8
9	62114.62
10	49148.01
11	59006.92
12	41132.41

	SHR	SHR-Glo1$^{+/-}$
CPM	59066.55 ± 334.23	58980.79 ± 2569.49
LM	47156.88 ± 448.08	50425.41 ± 8159.35

pAMPK

SHR　　SHR-*Glo1*$^{+/-}$

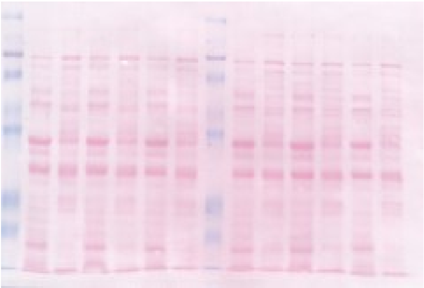

1 2 3 4 5 6 7 8 9 10 11 12

Sample number	Optical density
1	40318.06
2	35862.06
3	42801.22
4	37442.76
5	43881.32
6	36638.42
7	49631.96
8	35198.47
9	49474.39
10	37580.59
11	40105.35
12	33297.01

	SHR	SHR-Glo1$^{+/-}$
CPM	42333.53 ± 1491.81	46403.90 ± 4454.21
LM	36647.75 ± 645.35	35358.69 ± 1752.43

1, 3, 5, 7, 9, 11 – CPM

2, 4, 6, 8, 10, 12 – LM

B – Liver tissue samples

GLUT4

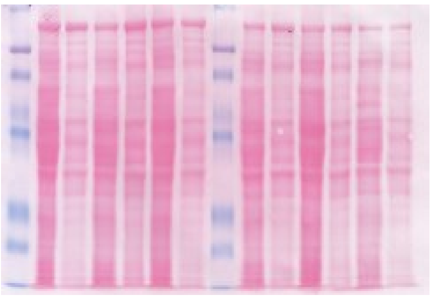

Sample number	Optical density
1	76592.74
2	43018.62
3	78757.18
4	52367.33
5	75669.31
6	57546.91
7	76100.96
8	59929.59
9	84030.4
10	48809.43
11	60580.11
12	35641.99

Average optical density

	SHR	SHR-Glo1$^{+/-}$
CPM	77006.41 ± 1294.11	73570.49 ± 9739.32
LM	50977.62 ± 6012.00	48127.00 ± 9927.11

AMPK

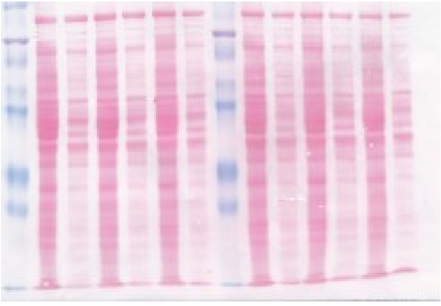

Sample number	Optical density
1	87457.33
2	41435.11
3	83166.75
4	44101.94
5	85499.57
6	43014.31
7	87531.8
8	42692.94
9	85563.85
10	48627.21
11	84276.68
12	43273.73

	SHR	SHR-Glo1$^{+/-}$
CPM	85374.55 ± 1753.85	85790.78 ± 1338.55
LM	42850.45 ± 1094.88	44864.53 ± 2671.09

pAMPK

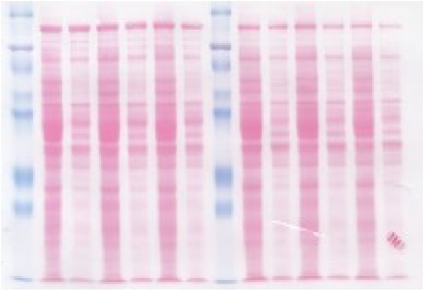

Sample number	Optical density
1	80645.93
2	40790.32
3	84377.75
4	43626.1
5	84883.17
6	43847.51
7	88459.95
8	45441.2
9	86759.53
10	45425.15
11	80289.02
12	37531.03

	SHR	SHR-Glo1$^{+/-}$
CPM	83302.28 ± 1889.63	85169.50 ± 1391.92
LM	42754.64 ± 3520.15	42799.13 ± 3725.11

1, 3, 5, 7, 9, 11 – CPM

2, 4, 6, 8, 10, 12 – LM

C – Fat tissue samples

GLUT4

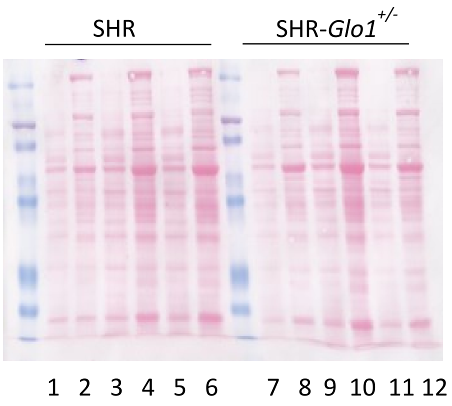

Sample number	Optical density
1	35435.31
2	48037.37
3	51571.15
4	90712.49
5	53397.81
6	92476.97
7	32427.83
8	59656.69
9	52540.44
10	100929.8
11	32792.43
12	69967

Average optical density

	SHR	SHR-Glo1$^{+/-}$
CPM	46801.42 ± 8071.58	39253.57 ± 9396.42
LM	77075.61 ± 20545.77	76851.16 ± 17538.74

AMPK

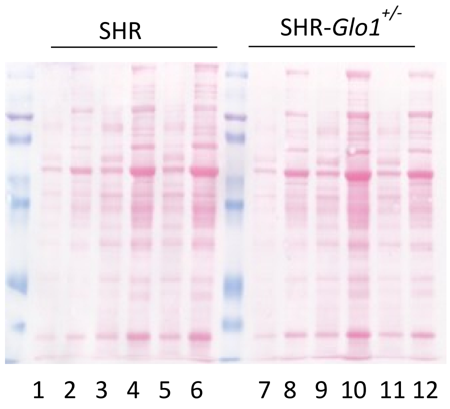

Sample number	Optical density
1	27723.14
2	33036.83
3	41416.85
4	65096
5	41029.09
6	68398.71
7	26996.87
8	38459.8
9	42031.19
10	79164.37
11	36236.36
12	50023.5

	SHR	SHR-Glo1$^{+/-}$
CPM	36723.03 ± 6365.85	35088.14 ± 6191.20
LM	55510.51 ± 15948.39	55882.56 ± 17126.23

pAMPK

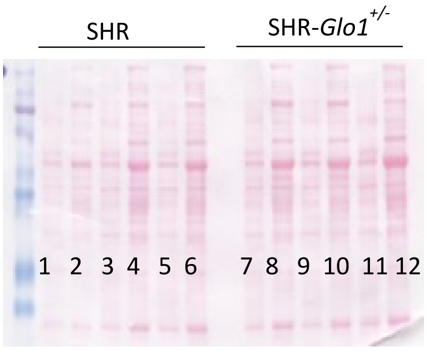

Sample number	Optical density
1	25574.77
2	30031.09
3	26615.26
4	46363.46
5	27456.55
6	48458.15
7	27547.99
8	46127.34
9	29298.35
10	46069.34
11	34274.15
12	55316.29

	SHR	SHR-Glo1$^{+/-}$
CPM	26548.86 ± 769.67	30373.50 ± 2849.24
LM	41617.57 ± 8237.39	49170.99 ± 4345.45

1, 3, 5, 7, 9, 11 – CPM

2, 4, 6, 8, 10, 12 – LM

Supplementary Materials

Examples of original images of immunoblots

Abbreviations:

St, molecular weight standard; *CPM*, crude plasma membranes; *LM*, light microsomal membranes; PNS, postnuclear supernatant; WAT, white adipose tissue

Fig. 1 – Levels of Glo1 in PNS from heart, liver and WAT of SHR and SHR-*Glo1*$^{+/-}$ rats

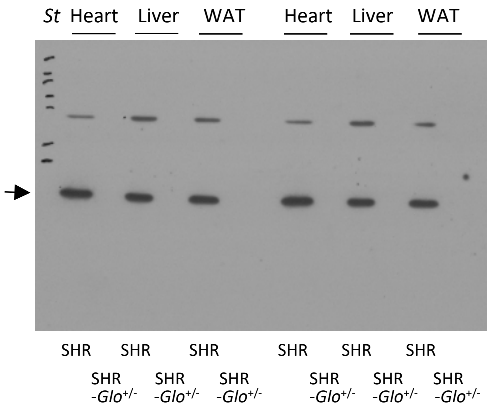

Fig. 2 – Markers of CPM and LM

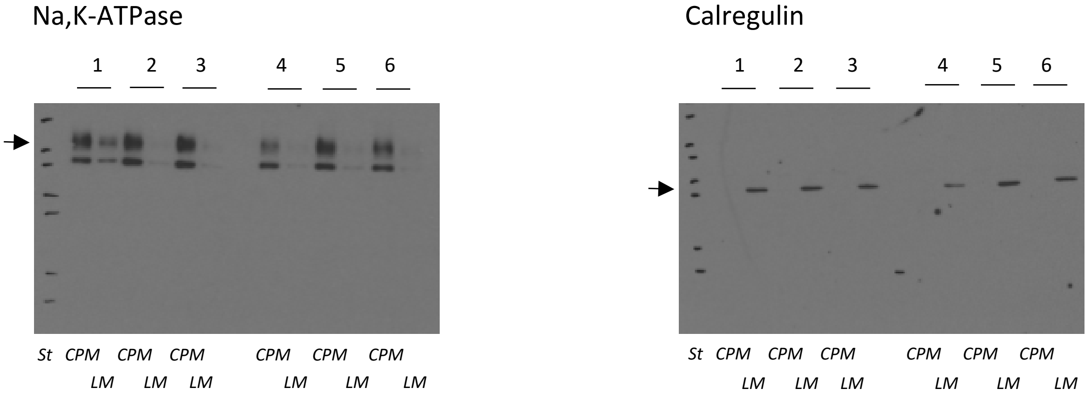

Fig. 3A – Heart tissue samples

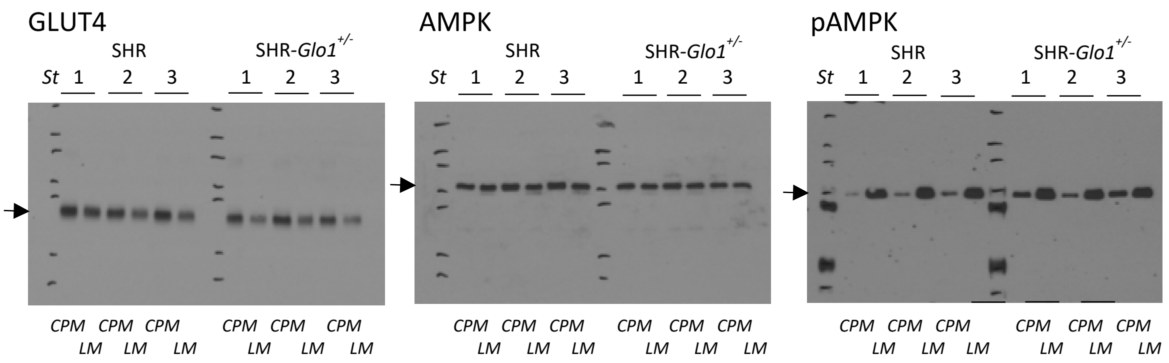

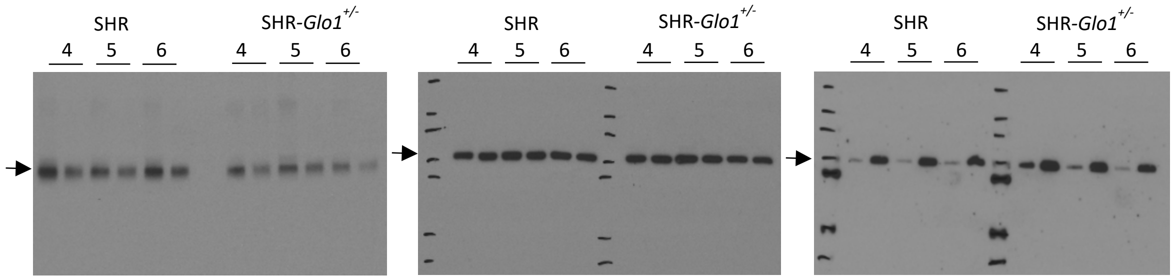

Fig. 3B – Liver tissue samples

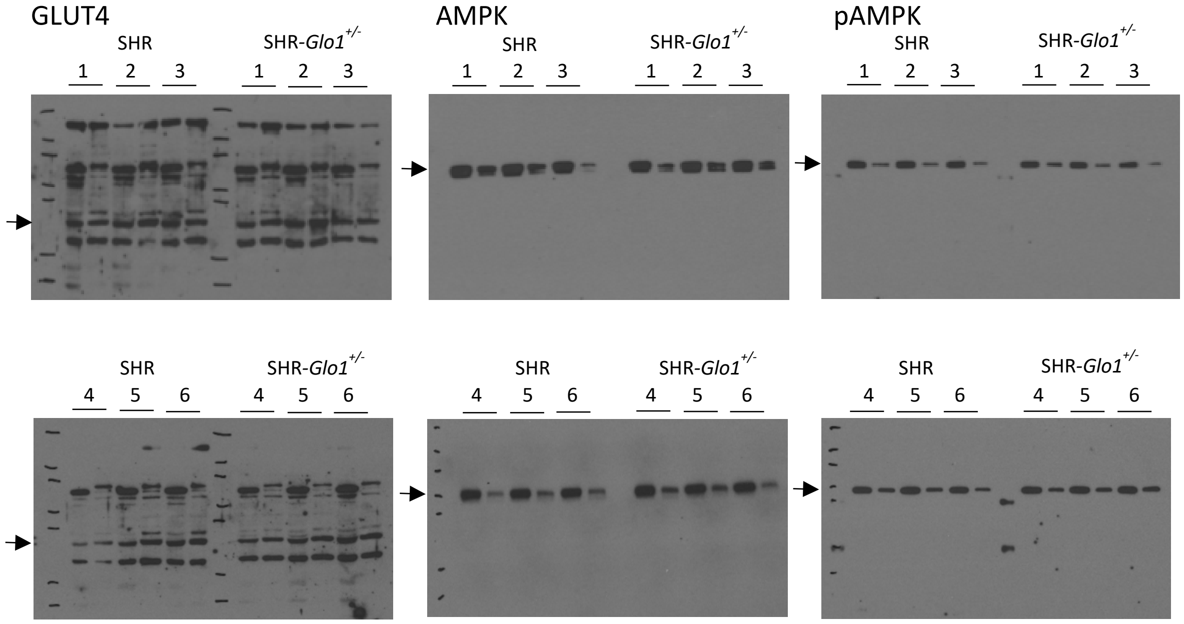

Fig. 3C – White fat tissue samples

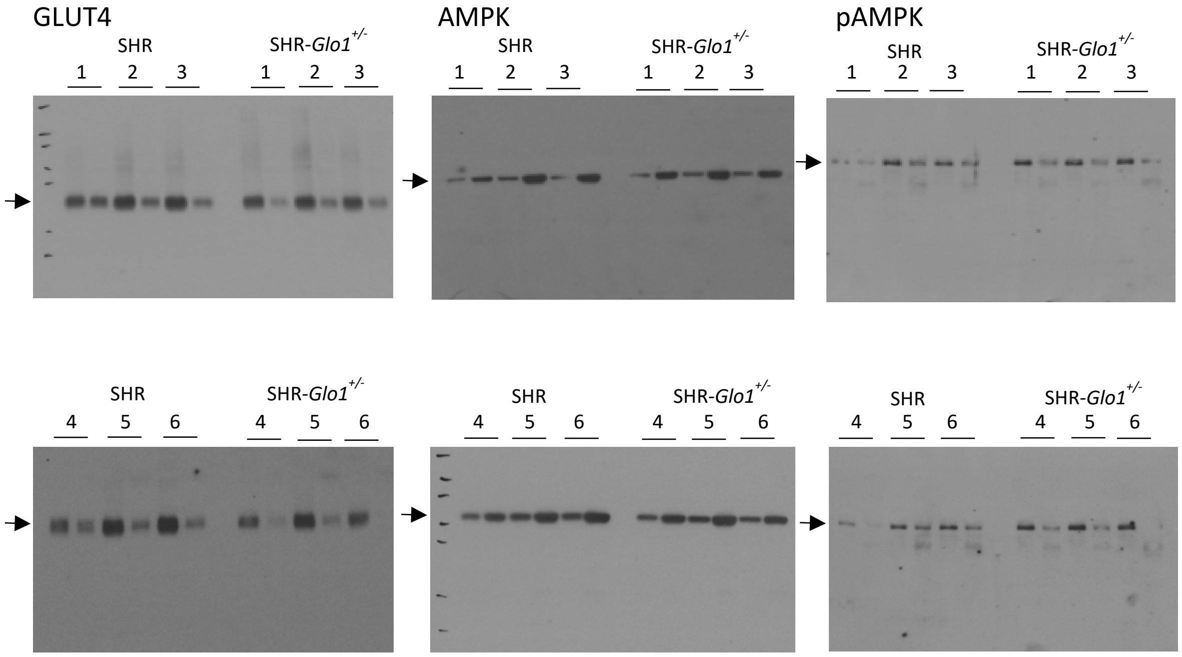

Supplement: Supplementary file 1 [file antioxidants-09-01179-s001.pdf]
